# The Effect of Ti/Ta Ratio and Processing Routes on the Hardness and Elastic Modulus of Porous TiNbZrTa Alloys

**DOI:** 10.3390/ma16237362

**Published:** 2023-11-27

**Authors:** Celia González-Guillén, Ghaith Al Hawajreh Kamel, Eduardo Degalez-Duran, Elizaveta Klyatskina, Muhammad Naeem, Liliana Romero-Resendiz, Gonzalo Gonzalez, Vicente Amigó Borrás

**Affiliations:** 1 Instituto de Tecnología de Materiales, Universitat Politècnica de València, Camino de Vera s/n, 46022 Valencia, Spain; celiagonzgui@gmail.com (C.G.-G.); galhkam@doctor.upv.es (G.A.H.K.); elkl1@upv.es (E.K.); 2 Departamento de Ingeniería Metalúrgica, Facultad de Química, Universidad Nacional Autónoma de México, Mexico City 04510, Mexico; degalezduranedu@gmail.com; 3 School of Metallurgy and Materials, University of Birmingham, Birmingham B15 2TT, UK; m.naeem@bham.ac.uk; 4 Instituto de Investigaciones en Materiales, Universidad Nacional Autónoma de México, Circuito Exterior S/N, Cd. Universitaria, A. P. 70-360, Coyoacán C.P. 04510, Mexico

**Keywords:** high-entropy alloy, porosity, blend element, mechanical alloying, mechanical properties, impulse excitation technique, powder metallurgy, casting

## Abstract

TiNbZrTa alloys are promising for multidisciplinary applications, such as refractory and biomedical purposes, due to their high thermal stability and non-toxicity. Hardness and elastic modulus are among the key features for their adequate industrial applications. The influence of porosity and Ti/Ta ratio were investigated on TiNbZrTa alloys produced by three different processing routes, i.e., (i) blend element and posterior press and sintering (BE + P&S); (ii) mechanical alloying with press and sintering (MA + P&S); and (iii) arc melting and casting. Porosity decreased in the following order: casting < MA + P&S < BE + P&S. The total porosity of alloys increased with increasing Ta contents, i.e., by lowering the Ti/Ta ratio. However, the Ti/Ta ratio did not considerably affect the bonding energy or the elastic modulus. Hardness was increased significantly in dense alloys compared to porous ones. However, porosity and Ti/Ta ratio did not show a clear trend in hardness among the porous alloys.

## 1. Introduction

High-entropy alloys (HEAs), also called multi-component or multi-principal element alloys, are constituted of four or more principal elements with nearly equimolar concentrations [1]. Although most of the work on HEAs has focused on mechanical properties [2], interesting works related to the magnetic, catalytic, or biomedical applications of HEAs have been reported as well [3,4,5]. Among the latter, the high-entropy TiNbZrTa (TNZT) alloy is garnering significant attention for its exceptional thermal stability at elevated temperatures and remarkable biocompatibility [6,7], attributed to the low cytotoxicity of its constituent elements, Ti, Nb, Zr, and Ta [8]. As a result, this alloy stands out as a promising material for applications in refractory and biomedical fields. Both applications converge on the principle of mechanical adequacy to secure their desired functionality: (i) the hardness should be high enough to avoid shear failure [9] during biomedical or refractory performance; (ii) the stiffness, i.e., elastic modulus, should be as close as possible to that of human bone to avoid stress shielding during biomedical performance [10,11] yet high enough to resist deformation during refractory applications.

Chemical composition and porosity are major parameters which affect stiffness and hardness. Different attempts to tune mechanical properties by minority alloying additions to the highly concentrated TNZT alloys have been reported, e.g., Fe [12,13,14], Si [12,13], O [12], Ag [14], Sn [14], etc. Variations of the base elements have also been studied, e.g., the Ti/Ta ratio [15,16]. So far, no systematic study of the effect of porosity on the mechanical properties of TNZT alloys has been reported. Similarly, the effect of chemical composition on their hardness and elastic modulus has not been well established.

Recently, a multitude of paradoxes of conventional metallic materials have been challenged when applied to HEAs [2]. This is mainly because of the effect of multiple principal elements in equiatomic or near equiatomic amount in HEAs, as opposed to the single principal element in conventional alloys [17]. Due to the multiple elements with different atomic radii in a solid solution, the lattice distortion in HEAs may influence the interatomic bonding strength.

From the above, the understanding of the elastic modulus and hardness in HEAs has also been challenged. The elastic moduli of HEAs, which are highly dependent on the bonding strength, may vary from conventional alloys. As different pairs of atoms may be found at similar separation distances in HEAs, multiple bond strength values are expected [18]. In addition to the unconventional behavior triggered by lattice distortion in the TNZT alloy, porosity is another factor that can also affect stiffness [18,19]. Regarding hardness, it is empirically correlated to the yield strength in conventional metallic materials. However, low plasticity as well as low strain-hardening rate has been reported for TNZT-based alloys [6,16]. As the strain-hardening rate determines the difference between yield strength and ultimate tensile strength, its tendency cannot be accurately characterized from the tensile test of TNZT alloys with low plasticity. The correlation of the tensile yield strength to hardness should be applied with caution [20,21]. Previous reports have shown that this conventional relationship is much higher than fracture strength in weak strain-hardened HEAs because of their premature fracture under uniaxial loading [21]. In addition to the above, chemical composition changes and the introduction of 3D defects, such as pores, may also affect the hardness of TNZT alloys.

In this work, we report comparative correlations to describe the effect of chemical composition and porosity on the hardness and elastic modulus of different TNZT alloys. Different powder metallurgy (PM) techniques and arc melting were used to produce materials with different porosities. This study will serve as a foundation for the future design of TNZT-based components for refractory and biomedical applications.

## 2. Materials and Methods

### 2.1. Processing of TNZT Alloys

The properties of powder materials used to produce the TNZT alloys are shown in Table 1. All of them were provided by Alfa Aesar. The chemical compositions of TNZT alloys explored in this study are given in Table 2.

#### 2.1.1. Powder Metallurgy Using the Blend Element Technique

The alloys produced using the blend element (BE) and posterior press and sintering (P&S) method are hereinafter referred to as BE + P&S samples. The chemical composition of alloys produced using the BE + P&S method are shown in Table 2. Powders with the desired compositions were mixed using a blender (Bioengineering Inversine 2L, Wald, Switzerland) in a closed vial for 30 min at 54 rpm. Blend elements were used before sintering to secure their homogeneous mixing. Approximately 8 g of the powders was compacted at 1000 MPa through a rectangular die with the dimensions of 32 mm × 12 mm × 6 mm. The specimens were sintered in a high-vacuum tubular furnace (Carbolite HVT 15/75/450, Chelmsford, England) at a pressure between 0.01 and 0.1 Pa. A two-step sintering process was performed as follows: (1) increasing the temperature at a speed of 5 °C min^−1^ until 800 °C and maintaining for two hours, and (2) increasing the temperature up to 1400 °C and maintaining the temperature for three hours. Posteriorly, the samples were furnace-cooled at 10 °C min^−1^.

#### 2.1.2. Powder Metallurgy Using Mechanical Alloying

The alloys produced using mechanical alloying (MA) and posterior P&S are hereinafter referred as MA + P&S samples. The chemical composition of alloys produced using this method are shown in Table 2. MA was performed in a planetary ball mill (Retsch model PM 400/2, Haan, Germany) at 350 rpm for 40 h, divided into work stages of 8 min on and 10 min off to improve the homogenization of the TNZT alloy after press and sintering (P&S). Standard stainless steel jars and chromium steel balls with a ball-to-powder weight ratio of 10:1 were used for milling, while 1 wt.% of stearic acid (C_18_H_36_O_2_) was used as a debinding agent to avoid powder agglomeration. The powders were then compacted using a hydraulic press (Metallkraft model WPP 50 M, Kristiansand, Norway) and a floating die of 1.8 cm3 at 1000 MPa for 10 s. The specimens were sintered in a high-vacuum tubular furnace (Carbolite HVT 15/75/450) at a pressure between 0.01 and 0.1 Pa with Ar atmosphere. Sintering was performed in three steps: (1) temperature increase at 5 °C min^−1^ until 800 °C and holding for one hour; (2) temperature increase up to 1400 °C and holding for three hours; and (3) furnace-cooling at 10 °C min^−1^ to room temperature.

#### 2.1.3. Arc Melting and Casting

The alloys produced using arc melting are hereinafter referred as casting or cast samples. The chemical composition of alloys produced using this method are shown in Table 2. Arc melting was carried out using an Edmund Bühler GmbH MA M-1 (Bodelshausen, Germany) under inert atmosphere to avoid the formation of oxides. The alloys were remelted six times to ensure homogeneity.

### 2.2. Microstructural Characterization

Cross-sections of the HEAs were subjected to standard metallographic preparation, i.e., grinding with SiC papers and polishing with an OP-S NonDry suspension of 0.04 μm particle size diluted in 20% H_2_O_2_. Microstructural observations were carried out using optical microscopy with a Nikon Eclipse LV100 (Tokyo, Japan). Pore size ranges were measured using digital micrograph software version 3.53.4137.0 from Gatan Inc (Pleasanton, CA, USA). considering at least 50 pores in each sample. Closed, open, and total porosities were estimated using the Archimedes method according to ASTM B962-17 [22]. Dry (*m_d_*), submerged (*m_s_*), and wet (*m_w_*) masses of the samples were measured on a KERN 770 precision balance with adapted clamping structure and counterweights. The open porosity percentage (*% P_o_*), closed porosity percentage (*% P_c_*), and total porosity percentage (*% P_t_*) were calculated based on Equations (1)–(7).

(1) $$V_{t\;}=\frac{m_{d}}{\rho_{d}}\;$$

(2) $$V_{o\;}=\frac{m_{w}-m_{d}}{\rho_{water}}\;$$

(3) $$V_{app\;}=\frac{m_{s}-m_{w}}{\rho_{water}}\;$$

(4) $$V_{c\;}=V_{app}-V_{t}-V_{o}\;$$

(5) $${\%P}_{o}=\frac{V_{o}}{V_{app}}\times 100\;$$

(6) $${\%P}_{c}=\frac{V_{c}}{V_{app}}\times 100\;$$

(7) $${\%P}_{t\;}={\%P}_{o}+{\%P}_{c}\;$$

where $\rho_{d}$ is the density of the dry mass, i.e., the theoretical density of the TNZT alloys (Table 2); $\rho_{water}$ is the density of water (1 g cm^−3^); $V_{t}$, $V_{o}$, $V_{app}$, and $V_{c}$ are the theoretical, open, apparent, and closed volumes, respectively. At least five measurements were averaged for each sample.

### 2.3. Mechanical Properties Evaluation

Microhardness was measured on the polished HEAs according to the ASTM: E384 using a Shimadzu Vickers HMV-2 microdurometer (Kyoto, Japan) by applying 3 N for 15 s. At least five measurements were obtained per sample. The elastic modulus was measured using the impulse excitation technique (IET, ATCP- Sonelastic). The software ATCP Sonelastic 3.0 was used to analyze the data.

## 3. Results and Discussion

**Figure 1:**
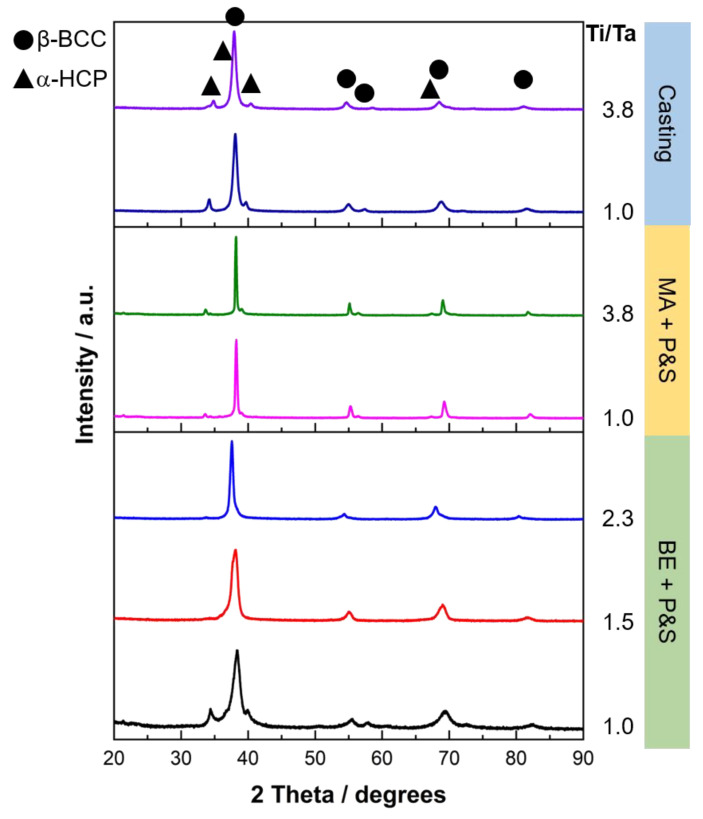
XRD patterns of the studied TNZT alloys with different Ti/Ta ratios.

**Figure 2:**
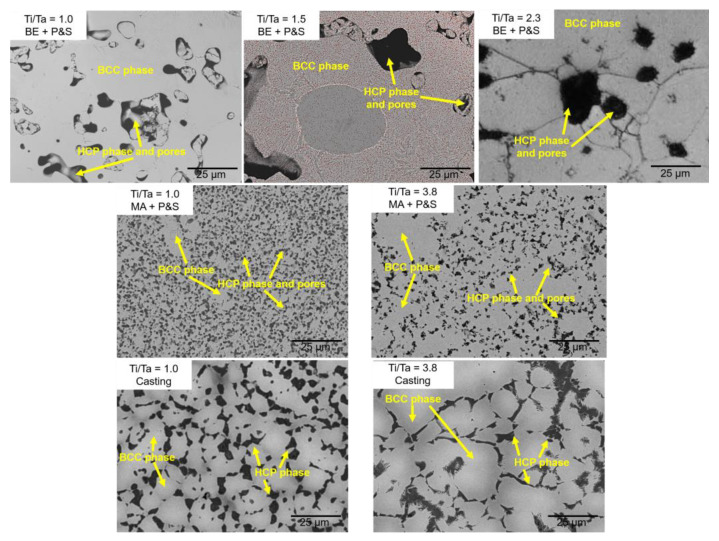
Micrographs showing the porosity of the studied TNZT alloys with different Ti/Ta ratios.

Figure 1 displays the XRD diffractogram of the studied TNZT alloys according to their Ti/Ta ratio. β-BCC and α-HCP phases were identified in all the samples. Figure 2 shows representative optical micrographs of the studied TNZT samples that allow for the observing of the present phases, as well as the morphology and size of pores and phases. A larger pore size was produced by the BE + P&S method (3–27 μm) compared to that by MA + P&S (0.5–3 μm) and cast alloys (0.5–1.0 μm). The difference in pore size is related to the processing parameters of each production route. Considering that BE was performed for 30 min without milling balls, while mechanical alloying was performed for 40 h with chrome-steel milling balls, the expected larger powder size from BE agrees with its larger pore size after sintering. As casting involves melting of the powders, negligible porosity was expected. The pores generated from the three routes showed rounded morphologies.

**Figure 3:**
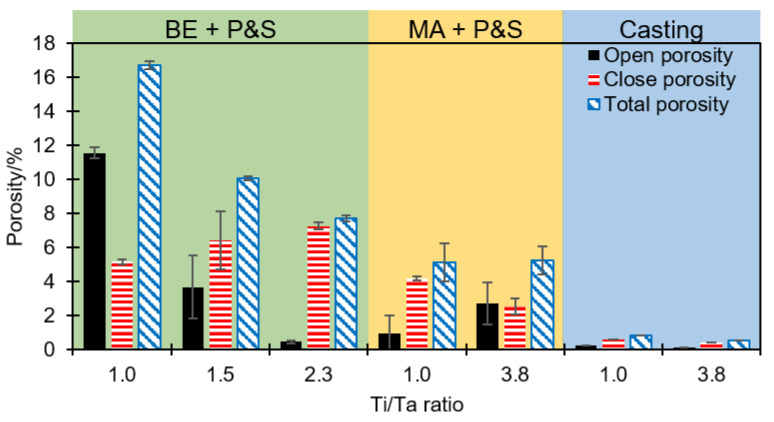
Open, closed, and total porosity as a function of the Ti/Ta ratio of each studied TNZT alloy.

Figure 3 shows the open (interconnected), closed, and total porosity of the studied alloys for each technique. BE + P&S shows the highest level of porosity compared to MA + P&S and casting. The solubility limit increment and welding phenomena are well documented from MA processing [23]. Possible higher atomic diffusion generated by MA could be responsible for the higher density compared to BE. BE enhances powder mixing and surface contact but does not promote metallic bonding in the TNZT alloys [16]. Casting generated the most compacted alloys with lower porosity content, being a result of the full melting process with expected highly activated atomic diffusion compared to that of PM methods. Compared to the sintering temperature, higher temperature during casting was expected to promote pore growth because of higher diffusion rates [24]. However, other parameters influencing atomic diffusion rates may also influence pore growth, e.g., cooling rate, pressure, and isothermal ramp duration, among others [24,25,26]. The cooling rate after sintering (10 °C min^−1^) is significantly lower than that from casting because of the air cooling after melting. The study of the effect of these parameters on the pore size requires future systematic investigation.

Porosity from BE + P&S increases, while the Ti/Ta ratio decreases. The Ti_25_Nb_25_Zr_25_Ta_25_, Ti_30_Nb_25_Zr_25_Ta_20_, and Ti_35_Nb_25_Zr_25_Ta_15_ samples showed total porosities of 16.7, 10.0, and 7.7%, respectively. Thus, porosity increased 2.2 times by decreasing the Ti/Ta from 2.3 to 1.0. This phenomenon is explained by the highest melting point of Ta (3020 °C, Table 1) compared to the other constituent elements (Ti: 1725 °C; Nb: 2477 °C; Zr: 1855 °C), which may cause reduced atomic diffusion during sintering. In addition, the expected larger powder size from BE compared to MA could reduce the surface contact of powders during sintering. These results are in agreement with the larger pore sizes produced by BE + P&S compared to the other two methods (Figure 2). Reducing atomic diffusion could also affect the bonding formation and bonding energy [27]. Reported BE + P&S TNZT alloys have shown elemental segregation. The lower the Ti/Ta ratio, the lower the atomic diffusion and grain size are [16].

The production processes with expected higher atomic diffusion, i.e., MA + P&S and casting, generated similar porosities regardless of the Ti/Ta ratio. The Ti_25_Nb_25_Zr_25_Ta_25_ and Ti_43.1_Nb_22.1_Zr_22.5_Ta_11.3_ produced by MA + P&S showed total porosities of 5.1 and 5.2%, respectively, while the cast Ti_25_Nb_25_Zr_25_Ta_25_ and Ti_43.1_Nb_22.1_Zr_22.5_Ta_11.3_ samples had total porosities of 0.8 and 0.5%, respectively. Considering that the P&S processes were completed in the same conditions, it is assumed that the difference in porosity is related to the expected lower atomic diffusion during the earlier stages of BE and MA. The elemental bonding generated by MA decreased the susceptibility of this route to Ti/Ta ratio compared to the BE. This agrees with the 3.2 times lower porosity in the Ti_25_Nb_25_Zr_25_Ta_25_ sample produced by MA + P&S than by BE + P&S. Thus, fabrication routes with expected higher atomic diffusion are less susceptible to the density of the constituent elements. Therefore, the independence of the processing method to the alloy density can be ordered in the following sequence: casting > MA + P&S > BE + P&S.

**Figure 4:**
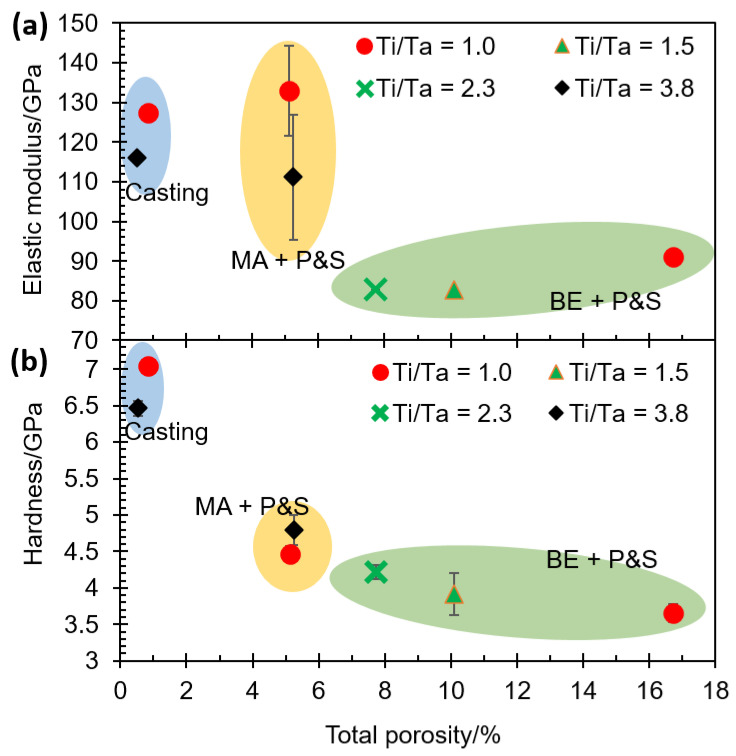
Mechanical properties of TNZT alloys: (**a**) elastic modulus and (**b**) hardness as a function of total porosity of the samples with different Ti/Ta ratios.

The abovementioned effect of atomic diffusion on bonding formation is expected to influence the elastic modulus. Figure 4a shows the elastic modulus as a function of the total porosity of alloys. It can be seen that the lower porosity generated a higher elastic modulus, i.e., the elastic modulus followed an inverse relationship with the total porosity. BE + P&S samples showed the lowest Young’s moduli values (82.7 to 91.0 GPa), followed by the MA + P&S (111.2 to 133.0 GPa), and then cast samples (115.9 to 127.3 GPa).

The experimental elastic moduli can be compared with the theoretical ones by using the lineal equation (Equation (8)) that Fryxell and Chandler proved on materials with pores fractions from 0.02 to 0.17 [28],

(8) $$E_{p}=E_{np}\left(1-aP\right)$$

where $E_{p}$ is the elastic modulus of the porous material; $E_{np}$ is the elastic modulus of the dense material; a is a constant related to the Poisson’s ratio of the matrix, and, here, a value of 1.9 is assumed [29]; and P is the pores fraction. The $E_{np}$ was taken from the cast Ti_25_Nb_25_Zr_25_Ta_25_ sample (127.3 GPa) where the porosity effect can be considered negligible for the mechanical properties. Thus, the theoretical elastic modulus of the Ti_25_Nb_25_Zr_25_Ta_25_ EB + P&S and MA + P&S samples is of 86.8 and 114.9 GPa, respectively. Theoretical values are 4.8 and 15.7% lower than the experimental values of the EB + P&S and MA + P&S Ti_25_Nb_25_Zr_25_Ta_25_ samples, respectively (91.0 and 133.0 GPa, respectively). Even though bonding energy is recognized as the main factor influencing the elastic modulus, the difference between theoretical and experimental values may be related to other secondary microstructural factors [30], such as the previously discussed lower atomic diffusion and possible elemental heterogeneous distribution from the PM methods compared to casting, which may also result in different phase percentages, or possible preferred crystallographic orientations (texture).

The lower Ti/Ta ratio promoted higher elastic modulus values. The elastic modulus in the BE + P&S samples increased 9.9% when increasing the Ti/Ta ratio from 1.0 to 2.3. An increment of 19.6% in the elastic modulus was observed when decreasing the Ti/Ta ratio from 3.8 to 1.0 in the MA + P&S samples. Samples produced by casting also showed an increment of 9.7% in the elastic modulus when decreasing the Ti/Ta ratio from 3.8 to 1.0. This implies that Ti/Ta ratio does not restrict the bonding strength, which is the main factor influencing the elastic modulus. Wolverton [31] has reported a direct correlation between the atomic radius of alloying elements and the bonding energy in Al alloys. Therefore, the main effect of lowering the Ti/Ta ratio, i.e., increasing high-density Ta (Table 1), was increasing the density of the alloys with a probable restriction of atomic diffusion during their processing. This is in good agreement with the highest porosity of the samples with the lowest Ti/Ta ratio (Figure 3).

The highest hardness was observed in the dense cast alloys (7.0 GPa) compared to the porous ones (3.6 to 4.7 GPa) which were produced using PM methods (i.e., BE + P&S and MA + P&S). The comparison of the PM-processed TNZT alloys with a Ti/Ta ratio of 1.0 showed a hardness decrement of 21% in the sample with 16.7% porosity (BE + P&S) compared to the one with 5.1% (MA + P&S) (Figure 4b). Due to the lower rigidity induced by pores, the hardness decreased with the total porosity. These results agree with the effect of porosity on hardness of multiple Ti alloys for biomedical purposes [32,33]. This decrement in hardness in the BE + P&S samples could also be related to their higher pore size (3–27 μm) in contrast to the MA + P&S samples (0.5–3 μm) (Figure 2). This may be related to the pore size-sensitive elastic recovery in porous materials that causes unpredictable behavior in load-bearing mechanical measurements, such as hardness [34,35]. Considering that the elastic modulus was measured by IET, the effect of elastic recovery may not be significant. However, this hypothesis needs to be confirmed by future systematic research on the effect of pore size on hardness, as its effect has not been well established so far in the literature [34].

Despite the widely reported local lattice strain discrepancy between neighboring cells due to a large atomic size mismatch in HEAs [2], the Ti/Ta ratio did not show an obvious effect on the hardness of the studied TNZT alloys. Hardness decreased to 6.9 and 13.2% with Ti/Ta ratio decrements of 2.8 and 1.3 in the MA + P&S and BE + P&S samples, respectively. However, hardness increased 8.3% in the cast sample with a Ti/Ta ratio decrement from 3.8 to 1.0. Considering that hardness depends mainly on microstructural features, the unclear tendencies of pore size and Ti/Ta ratio on hardness may be related to other microstructural features. Examples of the above are grain size, phase percentage, elemental distribution, or preferred crystallographic orientation. Some materials with low porosity, similar to the ones studied here, have reported a negligible effect of porosity on hardness [36].

The elastic modulus showed a strong dependence on porosity, while the hardness was just slightly impacted by porosity in TNZT alloys. This is related to the fact that elastic modulus is mainly dependent on the bonding energy, while hardness is mainly dependent on microstructural factors, such as grain size, phase content, grain boundary features, etc. [37]. Regarding processing routes, the close values of bulk elastic moduli achieved by casting and MA + P&S samples indicate similar interatomic bonding strength. The difference in mechanical properties between the three studied processing routes is given by the difference in porosity or Ti/Ta ratio. The former effect is reflected in the properties associated with the microstructure (i.e., hardness), while the latter effect is more significant in the properties related to the bonding energy (i.e., elastic modulus).

## 4. Conclusions

Three series of TiNbZrTa alloys with different Ti/Ta ratios were developed using the methods of blend element and posterior press and sintering (BE + P&S), mechanical alloying with press and sintering (MA + P&S), and casting. The effects of porosity and chemical composition on elastic modulus and hardness were compared among the samples produced by the three different routes. The main findings can be summarized as follows:

1. The processing methods were influenced by the high-density Ta contents (lowering the Ti/Ta ratio) and produced total porosity in the following order: casting (0.5–0.8%) < MA + P&S (5.1–5.2%) < BE + P&S (7.7–16.7%).
2. The lower the Ti/Ta ratio, the higher the total porosity of the alloys.
3. Elastic modulus showed a low susceptibility to the Ti/Ta ratio. Thus, Ti/Ta ratio did not show a strong effect on the bonding energy. Elastic modulus was higher for the routes with literature-based expected higher atomic diffusion (Casting: 115.9–127.3 and MA + P&S: 111.0–133 GPa) than for the BE + P&S samples (82.8–91.0 GPa). The close values of bulk elastic moduli achieved by casting and MA + P&S samples indicate similar strength of interatomic bonds. The lower porosity generated higher elastic modulus.
4. Hardness was boosted in the dense cast alloys (7.0 to 6.5 GPa) compared to the porous ones (3.6 to 4.8 GPa). A decrement in hardness was observed with increasing total porosity. However, pore size and Ti/Ta ratio did not show a clear tendency on hardness among the porous alloys.
5. The effect of porosity is mainly reflected in the properties associated with the microstructure (i.e., hardness), while the effect of Ti/Ta ratio is more significant in the properties related to the bonding energy (i.e., elastic modulus).

Systematic studies evaluating the influence of other microstructural parameters on the hardness and elastic modulus of TiNbZrTa alloys, such as phase percentage, grain size, elemental distribution, pore distribution and morphology, and preferred crystallographic orientation are recommended for future research.

## Figures and Tables

**Table 1:** Characteristics of raw powders used to produce the TNZT HEAs.

Raw Material	Purity/ wt.%	Average Particle Size/µm	Structure	Density/ g cm^−3^	Melting Point/°C
Ti	99.9	44	HCP	4.5	1668
Nb	99.8	1–5	BCC	8.5	2477
ZrH_2_	99.9	4.5–6.5	FCC	5.6	800
Ta	99.9	1–5	BCC	16.6	3020

**Table 2:** Chemical composition, Ti/Ta ratio, production route, and bulk theoretical density of the TNZT alloys.

Chemical Composition/at.%	Ti/Ta Ratio	Production Route	Density/g cm^−3^
Ti_25_Nb_25_Zr_25_Ta_25_ (equiatomic)	1.0	BE + P&S	8.9
Ti_30_Nb_25_Zr_25_Ta_20_	1.5	BE + P&S	8.3
Ti_35_Nb_25_Zr_25_Ta_15_	2.3	BE + P&S	7.7
Ti_25_Nb_25_Zr_25_Ta_25_ (equiatomic)	1.0	MA + P&S	8.9
Ti_43.1_Nb_22.1_Zr_22.5_Ta_11.3_ (equimassic)	3.8	MA + P&S	7.2
Ti_25_Nb_25_Zr_25_Ta_25_ (equiatomic)	1.0	Casting	8.9
Ti_43.1_Nb_22.1_Zr_22.5_Ta_11.3_ (equimassic)	3.8	Casting	7.2

## Data Availability

The data presented in this study are available on request from the corresponding author.
